# ‘Out of the Tropics’ Sheds Light on Latitudinal Gradients in Clade Ages of Climbers, China

**DOI:** 10.1002/ece3.71324

**Published:** 2025-04-18

**Authors:** Mingfei Zhao, Hongbo Li, Yuhang Wang, Yuan Jiang, Muyi Kang, Kaixiong Xing

**Affiliations:** ^1^ Ministry of Education Key Laboratory for Ecology of Tropical Islands, College of Life Science Hainan Normal University Haikou China; ^2^ State key Laboratory of Earth Surface Processes and Resource Ecology, Faculty of Geographical Science Beijing Normal University Beijing China; ^3^ Institute of Environment and Sustainable Development in Agriculture Chinese Academy of Agricultural Sciences Beijing China; ^4^ Research Institute of Wetland Chinese Academy of Forestry Beijing China; ^5^ Beijing Key Laboratory of Wetland Services and Restoration Chinese Academy of Forestry Beijing China

**Keywords:** clade age, climbing plant, cold tolerance, liana, niche conservatism, out of the tropics hypothesis

## Abstract

We aim to test hypotheses on the patterns of clade age of climbing plants under climatic variations along the latitudinal gradients in China. Specifically, we uncover their general patterns of mean family age (MFA) and their climatic drivers. We evaluate the extents to which both the tropical niche conservatism hypothesis (TNC) and the out of the tropics hypothesis (OTT) can account for the MFA of climbing plants, respectively. A dataset including 2487 climbing species was used to quantify geographical patterns of MFA across China. Spatial regression analyses with information‐theoretical multi‐model selections were performed to estimate the importance of climatic variables. There were generally increasing trends of MFA from low to high latitudes for all types of climbers. For woody climbers, MFA was negatively correlated with minimum temperature and annual mean precipitation but positively correlated with seasonal temperature and precipitation, and was mostly influenced by mean temperature of the coldest quarter. For herbaceous vines, the MFA pattern showed relatively insignificant correlations with all the climatic variables. Our results highlight that the OTT hypothesis offers a promising explanation for the latitudinal MFA gradients of climbers in China (especially for woody climbers), which turn out to be contrary to the TNC predictions.

## Graphical abstract

The first survey concerning the geographical patterns of climbers in regional scale (in China). The mean family ages (MFA) of climbers and trees showed opposite trends with increasing latitude. The increasing MFA of woody climbers with increasing latitude could be effectively explained by the ‘out of the tropics hypothesis’.

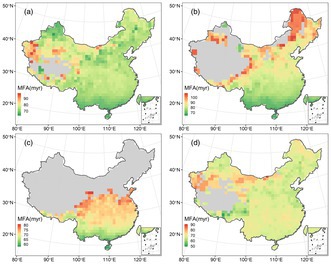

## 1 Introduction

The latitudinal diversity gradient (LDG), the increase of species diversity from the poles to the tropics, is one of the most pervasive patterns on Earth (Brown 2014; Mittelbach et al. 2007; Tordoni et al. 2024; Zhang et al. 2022). Although various empirical research involving both flora and fauna have endeavoured diligently to make explanations for LDG, the underlying mechanisms have not yet been universally understood (Brodie and Mannion 2023; Hillebrand 2004; Pontarp et al. 2019; Roy et al. 2000). It is well known that evolutionary (e.g., speciation, extinction, migration) and ecological (e.g., abiotic filters, biotic interactions) processes are two main critical drivers jointly shaping diversity patterns (Fine 2015; Wiens and Donoghue 2004; Wiens and Graham 2005). However, the imprints of evolutionary history had not received enough attention until rapidly developing big data became available (e.g., phylogenetic, molecular and paleontological data) (Currie et al. 2004; Kissling et al. 2012; L. Warren et al. 2014). Multiple lines of evidence have proved that ecological traits, especially of frost and drought tolerance, are phylogenetically conserved (Qian et al. 2021; Sklenář et al. 2023; Zanne et al. 2014). Therefore, seeking the relationships between clade age and ecological traits is another potential evolutionary perspective from which underlying mechanisms of biodiversity patterns can be explored (Mittelbach et al. 2007; Rangel et al. 2018).

The tropical niche conservatism hypothesis (TNC), from a combined perspective of both evolutionary and ecological processes, offers potential mechanisms underlying LDG (Qian et al. 2017; Wiens et al. 2006). Specifically, TNC posits that many extant clades originated in the tropics because in ancient times extensive land area (including high latitudes) was under tropical climates (i.e., with high temperature and high moisture) (Jansson et al. 2013) and temperate environments did not arise until global cooling initiated in the Eocene (Liu et al. 2009). The climatic cooling events triggered the retreat of most of the high‐latitude lineages to lower latitudes, leaving only a minority of lineages with adaptation to frost that managed to survive colder conditions (Economo et al. 2018). However, compared with niche shifting to preferred environments, it involves more difficult and progressive processes for species to evolve frost tolerance (Zanne et al. 2014), indicating that tropical areas are supposed to harbor deeper‐rooted clades (Hawkins et al. 2014; Qian 2014). Hence, it is predicted by the TNC hypothesis that tropical regions should be dominated by older clades, whereas extratropical regions are occupied by members of more recently derived clades that have undergone niche evolution, and that accordingly the clade age of species in an assemblage is younger as temperature or moisture decreases (Qian 2016).

Reversed latitudinal trends of species clade age are also logically predicted by another hypothesis—the ‘out of the tropics hypothesis’ (OTT). It suggests that the tropics act as the cradles of biodiversity (i.e., regions with high speciation rates) from which most clades have originated (Jablonski et al. 2006), and some old clades had ‘pumped out’ to higher latitudes through continuous species dispersal before the periods of global cooling and had somehow managed to survive there (Jablonski et al. 2006; Qian and Ricklefs 2016). However, the diversification of clades having invaded the extratropics is generally believed to be much slower than that of their tropical relatives (Qian and Ricklefs 2016), whereas in the tropics, not only have the representatives of most old families been preserved, but the diversification of new taxa is accelerated as well (Arita and Vázquez‐Domínguez 2008). Thus, the OTT hypothesis predicts that the tropics harbour both old and young clades while extratropical regions are less able to evolve younger clades owing to lower diversification rates (Qian and Ricklefs 2016), therefore the average clade age exhibits an increasing latitudinal gradient.

Climbers, with various types of scandent traits, can effectively strengthen their ability to compete with self‐supporting plants (e.g., trees or shrub) for local resources (e.g., light and space) (Gentry 1991). For instance, thinner stems with larger amounts of leaves supported per stem diameter make it more efficient for climbers to transport water and nutrients to distant leaves (Schnitzer 2018). However, the very same traits may incur vulnerability to coldness‐ or drought‐induced embolism (Couvreur et al. 2015; Jiménez‐Castillo and Lusk 2013), which can largely restrict their distribution to the extratropics (Ewers and Fisher 1991). Therefore, species with scandent habits are potentially as ideal subjects as, if not better than, trees, for investigating relationships between clade age and climatic factors in an attempt to uncover the underlying LDG mechanisms.

Since, at the rank of family, taxa traits are often strongly conserved and intra‐familial variation in traits is ignored, family age is considered an appropriate clade age with which to test the relevant hypotheses (Latham and Ricklefs 1993). Previous studies merely focused on the relationships between family age of angiosperm trees and climate variables, most of which found concordant results with predictions by the TNC. For example, research on North American forest communities found that angiosperm trees from older families prevail in lower latitudes whereas higher latitudes are dominated by younger ones (Hartmann et al. 2014; Qian et al. 2017). Similarly, the MFA of angiosperm trees was positively correlated with minimum temperature and annual precipitation both in South America and China (Qian et al. 2017; Qian and Chen 2016). However, what should be noted is that contrary tendencies which supported the predictions by the OTT were found (Qian 2014). It is thus admitted that discussions on LDG for terrestrial plants were mostly confined to tree species, and that TNC has failed to provide a consensus answer to the patterns of their clade age.

Here, we attempt to explore the relationships between mean family age (MFA) and climatic variables for angiosperm climbing plants in China in order to verify the hypotheses on large‐scale species diversity patterns. Specifically, we aim to verify the following predictions: (1) when the TNC hypothesis works, a positive relationship would be expected between MFA and temperature (or precipitation); (2) otherwise, MFA would decrease with temperature (or precipitation) and support the OTT hypothesis.

## 2 Materials and Methods

### 2.1 Data Collection

All of the climbers were confirmed based on their descriptions in Flora of China (http://www.iplant.cn/frps). Botanical nomenclature was standardised according to The Plant List (ver. 1.1, http://www.theplantlist.org/). Cultivated and exotic species were excluded. Taxa below species level (e.g., varieties and sub‐species) were integrated into their corresponding species. Consequently, a total of 1391 lianas, 437 scandent shrubs and 658 herbaceous vines were included in our study.

County‐level species distribution data for climbing plants in China were extracted from Flora Reipublicae Popularis Sinicae (FRPS), and converted into grid maps projected to Albers equal‐area grid cells with a resolution of 100 km × 100 km, which decreased the effects of potential sampling incompleteness (Xu et al. 2018). We also supplemented the location records in our study using specimen records in the National Specimen Information Infrastructure (NSII; www.nsii.org.cn). To further improve the accuracy of the species distribution data, we used the altitudinal range (obtained from data sources mentioned above) of each species to rectify its geographical range (Zhang et al. 2015). Specifically, the whole research region was firstly rasterized at the same resolution as the species distribution map was, and then the gridded research region and the 30 m × 30 m digital elevation model obtained from the United States Geological Survey (http://reverb.echo.nasa.gov/reverb/redirect/wist) were overlapped to calculate the altitudinal range of each grid cell. Then, county‐level distributions of species were overlaid on the gridded map of China, and species were removed if their altitudinal range was inconsistent with the elevation range of a grid. Finally, grid cells with no less than five species and where no less than 50% of the land area was situated within the border of China were retained to achieve higher reliability and better visualisation. As a result, the climbers as a whole and their subcategories including lianas, scandent shrubs and herbaceous vines, occupied 872, 651, 361 and 822 cells, respectively.

The crown family age data were derived from Zanne et al. (2014) (see Table S1). Each species in each grid was assigned the age of the family to which it belongs, and the mean family age (MFA) of each grid was calculated as the mean value of family ages of all species within the grid (Hawkins et al. 2014).

Mean temperature of the coldest quarter (MTCQ), mean annual precipitation (MAP), temperature seasonality (TSN) and precipitation seasonality (PSN) obtained from the WorldClim database (http://www.worldclim.org/) were selected as climatic variables, which were widely accepted as pivotal climatic correlations to MFA (Qian et al. 2017). Furthermore, pairwise correlations between the four variables were all less than 0.7 in our study, which indicated that there was no serious multicollinearity among the variables.

### 2.2 Data Analyses

We first used ordinary least squares (OLS) linear regressions to test relationships between MFA and climatic variables. Since significant spatial autocorrelation can incur inflation of type I errors in the residuals of the OLS model, we then performed spatial simultaneous autoregressive error models (SAR_err_) (Dormann et al. 2007). SAR_err_ models additionally add a spatial autocorrelation structure of a given dataset and assume that the autoregressive process can only be found in the error term (Kissling and Carl 2007). The spatial weight matrix in each SAR_err_ model was defined by second‐order neighbourhood and row‐standardised coding scheme (Xu et al. 2018) which consisted of zeros in the diagonal line and weights for the neighbouring locations in the off‐diagonal positions. Values of Akaike information criterion (AIC) were directly provided for SAR models and model fits (*R* ^2^) were assessed with pseudo‐*R* ^2^ values calculated as the squared Pearson correlation between the predicted values and the observed ones (Kissling and Carl 2007). Moran's *I* index was calculated to compare the residual autocorrelations in OLS models and SAR_err_ models, which showed that SAR_err_ models effectively eliminated the spatial autocorrelation (Figure S1).

To quantify the performance of each explanatory variable included in the models, we also assessed all subset models of SAR_err_ (Anderson and Burnham 2004). Concretely, the Akaike weight for each model was calculated as the inverse AIC value, which was then used to calculate the averaged standardised regression coefficients across all models. Next, the importance value of each variable was calculated as the sum of the weights of all models containing the variable. Then, the pseudo‐*R* ^2^ of each subset model was calculated in the same way that it was in the full model. Finally, the unique contribution of each variable was presented as the difference between the pseudo‐*R* ^2^ of the full models and that of the SAR_err_ models without the variable.

To improve the linearity and normality of residuals in the models, MAP was square‐root‐transformed. Modified *t*‐tests were used to assess the statistical significance accounting for spatial autocorrelation when exploring bi‐relationships between MFA and each climatic variable (Dutilleul et al. 1993). All statistical analyses were performed in R software 4.3.1 (The R Foundation for Statistical Computing, https://www.r‐project.org/). SAR models were implemented by the package ‘spdep’ (Bivand et al. 2014), and modified *t*‐tests by the package ‘SpatialPack’ (Osorio and Vallejos 2019).

## 3 Results

**FIGURE 1:**
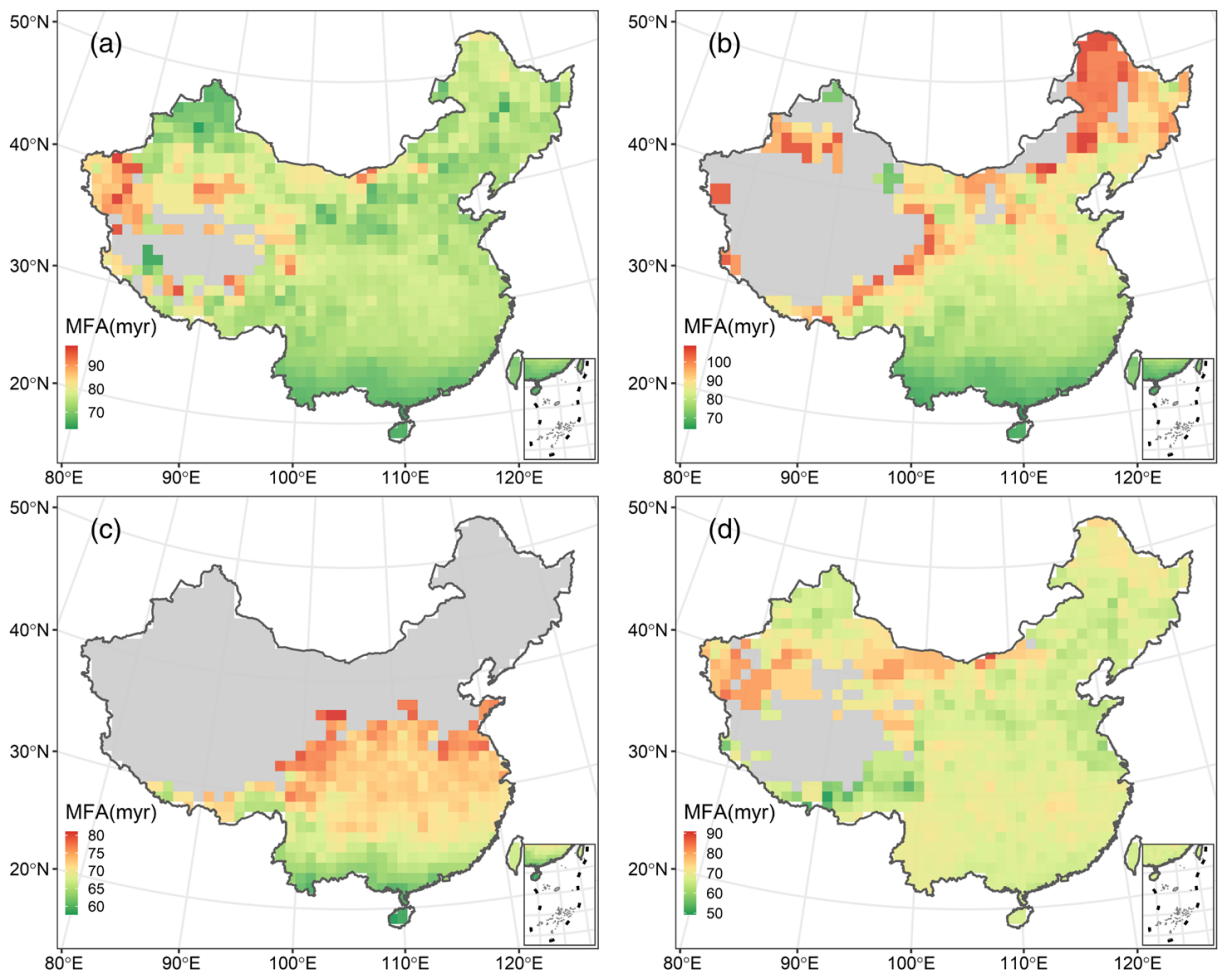
The spatial patterns of MFA for all climbers (a), lianas (b), Scandent shrubs (c) and herbaceous vines (d) in China exhibited in 100 km ×100 km grid cells. Only cells with richness ≥ 5 are shown for visual purpose.

The climber families in China included in this study covered large age intervals, ranging from *c*. 17 to *c*. 123 million years (myr) for lianas, from *c*. 32.9 to *c*. 92.3 myr for scandent shrubs, and from *c*. 30 to *c*. 112 myr for herbaceous vines (Table S1). Ignoring significance levels, the MFA of all climbers as well as three subcategories was positively correlated with latitude (all climbers, *r* = 0.222, *p* = 0.336; lianas, *r* = 0.775, *p* = 0.012; scandent shrubs, *r* = 0.865, *p* = 0.001; vines, *r* = 0.161, *p* = 0.379). In parallel, grid‐cell MFA demonstrated consistently increasing latitudinal trends (Figure 1). MFA patterns varied significantly between the tropics and extra‐tropical regions for woody climbers (i.e., lianas and scandent shrubs, Figure 1b,c), since most of the grids they occupied in temperate areas showed higher MFA values than those in tropical and subtropical zones. MFA for all climbers (Figure 1a) and herbaceous vines (Figure 1d) also exhibited northward and/or northwestward ascending trends, though not as distinct as in woody climbers.

**FIGURE 2:**
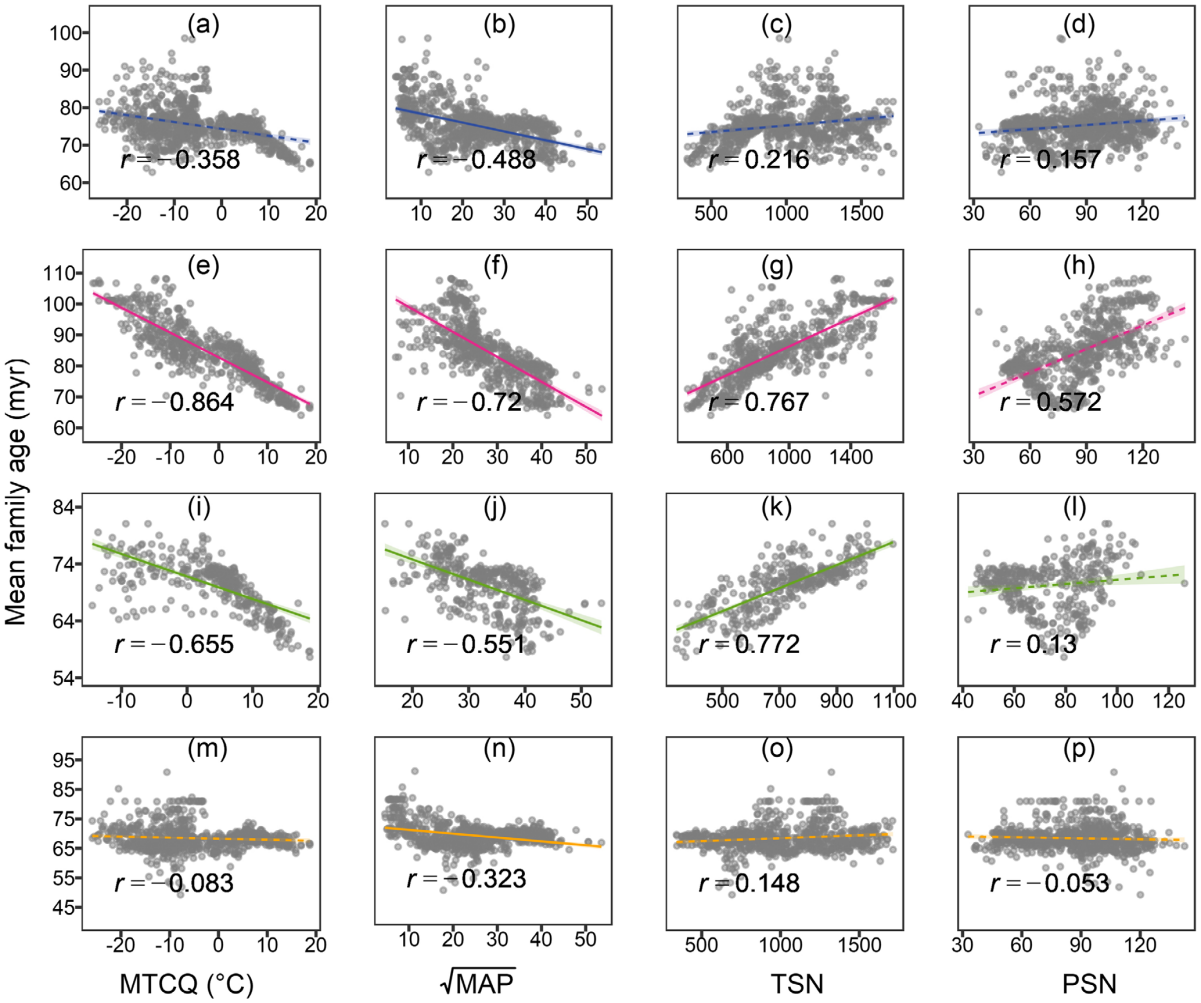
Relationships of MFA (million years, myr) with climate variables for all climbers (a–d), lianas (e–h), scandent shrubs (i–l) and herbaceous vines (m–p) in China. MAP, mean annual precipitation (square‐root‐transformed); MTCQ, mean temperature of the coldest quarter; PSN, precipitation seasonality; TSN, temperature seasonality. Ordinary least squares linear regressions are fitted to the data based on cells with species richness ≥ 5. Solid lines indicate significant results (*p* < 0.05); and dashed lines indicate insignificant results (*p* ≥ 0.05). Shaded areas represent 95% confidence intervals. *r*: Pearson coefficient tested by the modified *t*‐test.

Similar linear trends in the climbers' MFA were also shown along each environmental gradient (Figure 2). For all species and the three subcategories, MFA decreased with MTCQ and MAP but increased with TSN and PSN (Figure 2; Table 1), although the relationships for all species as a whole and for herbaceous vines alone were relatively insignificant (Figure 2a–d,m–p; Table 1), indicating a relatively homogeneous distribution in the clade age of herbaceous climbers. The relative importance of the explanatory variables on patterns of MFA was largely consistent among sub‐types (Table 1), with the temperature‐related factors (i.e., MTCQ and TSN) always presenting stronger correlations with MFA than moisture variables (i.e., MAP and PSN) did, with higher absolute regression coefficients, summed Akaike weights, *R* ^2^ and unique‐*R* ^2^.

**TABLE 1:** Results of spatial simultaneous autoregressive error (SAR_err_) models describing the relationships between four explanatory variables and MFA of climbing plants in China.

Variables	All climbers (*n* = 872, pseudo‐*R* ^2^ = 0.610)				Lianas (*n* = 651, pseudo‐*R* ^2^ = 0.694)				Scandent shrubs (*n* = 361, pseudo‐*R* ^2^ = 0.721)				Vines (*n* = 822, pseudo‐*R* ^2^ = 0.082)			
	Coef_avg_	Weights	*R* ^2^	*R* ^2^_unique_	Coef_avg_	Weights	*R* ^2^	*R* ^2^_unique_	Coef_avg_	Weights	*R* ^2^	*R* ^2^_unique_	Coef_avg_	Weights	*R* ^2^	*R* ^2^_unique_
MTCQ	−0.220*	0.895	0.123	0.000	−0.511***	1.000	0.698	0.128	−0.308**	0.997	0.44	−0.308**	0.044	0.295	0.007	0.017
MAP	−0.237	0.649	0.240	0.076	−0.031	0.293	0.444	0.001	−0.008	0.266	0.311	−0.008	−0.223	0.657	0.130	0.063
TSN	0.252*	0.76	0.042	−0.061	0.182**	0.899	0.507	0.004	0.504**	1.000	0.589	0.504**	0.211	0.647	0.028	−0.047
PSN	−0.075	0.407	0.023	0.013	0.109*	0.813	0.258	−0.012	0.150**	0.954	0.021	0.150**	0.065	0.356	0.001	−0.029

*Note:* *N*, in parentheses shows the number of grids occupied by each growth form; pseudo‐*R* ^2^, the maximum model fit of the full model; Coef_avg_, averaged standardized regression coefficients obtained from all models containing the focal variable; Weights, summed Akaike weights of all models containing the focal variable; *R* ^2^, squared Pearson correlation coefficients between non‐spatial trends of single‐predictor SAR_err_ model and observed values; Unique‐*R* ^2^, difference between the pseudo‐*R* ^2^ from the global model and that from the model without the focal predictor. We only showed cells with more than five species to avoid unstable results.

Abbreviations: MAP, mean annual precipitation; MTCQ, mean temperature of the coldest quarter; PSN, precipitation seasonality; TSN, temperature seasonality.

* *p* < 0.05.

** *p* < 0.01.

*** *p* < 0.001.

## 4 Discussion

### 4.1 Climatic Predictions of TNC and OTT for Climbers in China

We found that in northern China climbers had larger MFA values (i.e., higher proportion of species from old clades) than in tropic climates (Figure 1), and that MFA was negatively associated with temperature and moisture but positively with the seasonality indices (Figure 2, Table 1). To our knowledge, this is the first report concerning the geographical patterns of clade age for angiosperm climbers on a regional scale, and what we have discovered is considered atypical since it is almost contrary to the predictions of the TNC hypothesis, which has been supported by a majority of empirical data of angiosperm (primarily woody) species. For instance, studies concerning angiosperm tree assemblages from both China and The United States found decreasing latitudinal trends in MFA and strikingly positive relations between MFA and minimum temperature of the coldest month, which were in line with the TNC predictions (Hawkins et al. 2014; Qian and Chen 2016). Similarly, from the perspective of elevation, supporting evidence from woody and herbaceous assemblages located in a typical mountain slope of a temperate area in China was reported (but see (Qian et al. 2017; Zhao et al. 2018)). By contrast, our findings concerning climbing plants mainly matched what the OTT hypothesis predicts, indicating the possibility that OTT‐related mechanisms may play a dominant role in biogeographical processes for the climber clades, such as long‐distance dispersal and local diversification. We therefore suggest the following explanations for the seemingly contradictory observed patterns of clade age among climbing plants and other growth forms.

Basically, the biogeographical history for many clades reflects the major theme that niche conservatism interacts with functional trait evolution, which appears in diversified forms and in different hierarchies of scale (Wiens and Donoghue 2004). According to the TNC hypothesis, since most of the clades originated from the tropics, they are less able to cope with contrasting habitats with freezing temperatures, extreme droughts or dramatical climate oscillations (Wiens et al. 2010; Wiens and Graham 2005). The degree of difficulties the clades have to go through in niche evolution is supposed to be linked with large‐scale climate gradients throughout the globe and ultimately leaves relics in the divergences of clades derived from tropical regions and in the species range expansion to the extratropics (Wiens and Donoghue 2004). Therefore, the TNC hypothesis assumes that mean clade age will demonstrate a decreasing trend along latitudes and a positive correlation with temperature and precipitation (Qian and Ricklefs 2016; Wiens and Donoghue 2004).

On the other hand, OTT emphasizes the most radical parameters forging the global LDG, including rates of speciation, extinction and net immigration (Jablonski et al. 2006). Aside from the widely recognized views that the tropics are much more likely to act as both cradles (places with high diversification rates) and museums (places with low diversification rates) than temperate areas (Fischer 1960; Mittelbach et al. 2007), it is also underlined that tropical taxa retain a positive emigration rate on average, namely that some members from relatively old clades have been expanding their ranges into higher latitudes over time without losing their initial tropical distributions (Jablonski et al. 2006). The farther their dispersal to the tropics, the lower their diversification rates would be, and hence the earlier the old clades would have colonized the extratropical areas. Consequently, the tropics would harbor both old and young taxa while higher latitudes relatively lack younger clades (Qian and Ricklefs 2016; Stevens 2006). Collectively, it is reasonable to deduce a latitudinal decreasing gradient of net diversification rate fundamentally underpinned by global drivers such as energy and water, which would ultimately invoke reversed latitudinal trends and contradictory relationships between climatic factors and clade ages. These inferences were also demonstrated (Weir and Schluter 2007), who found that the tropics hold greater variance in ages of avian species than higher latitudes do.

In respect of our results, the seemingly ‘abnormal’ inclinations may not clash with the TNC hypothesis, but actually imply other OTT‐linked processes which are possibly so remarkable that they have masked the influence of TNC and have ultimately dominated the latitudinal family age gradients in the climbing plant groups. In fact, several old lineages (including early climbers) had managed to break through evolutionary limitations from adverse habitats (such as coldness) before their foray into higher latitudinal and altitudinal areas (Zanne et al. 2014). For example, in China, some old climber families with tropical affinity have succeeded in colonising temperate regions (e.g., species in Celastraceae, Smilacaceae, Vitaceae (aged c.61, c.81 and c. 117 Ma respectively)). It is reported that, to avoid suffering from harsh periods, some Vitaceae members are specialised in emptying xylem at the beginning of winter and refilling it prior to spring budding, and some species in Celastraceae are able to reproduce new xylem each year just the same way as temperate ring‐porous trees (Tibbetts and Ewers 2000). Others have found similar phylogenetic age patterns of tree species, indicating that, limitation to climbers aside, our study is not an isolated one. For example, an anti‐TNC pattern of MFA was discovered for trees in Chile, in which species from the oldest families prevailed in the mid to high latitudes whereas those from younger families dominated the lower latitudes (Segovia et al. 2013). According to them, one of the possible explanations for this pattern could be that some species from older families may have previously acquired adaptations to coldness before they spread to higher latitudes, while another interpretation suggested that the extreme aridity combined with the establishment of the South American Arid Diagonal may have promoted the evolutionary processes in situ associated with drought‐related adaptations (Segovia et al. 2013). In addition, there are also elevational studies conducted within tropical mountain areas that are assumed to partially provide clues for OTT (Qian 2014, 2016).

### 4.2 The Different Patterns of Mean Family Age Among Growth Forms of Climbers

We found a derivative geographical pattern and neutral correlations with climatic predictors displayed by the MFA of herbaceous vines (see Figure 1d, Figure 2m–p, Table 1). It possibly reflected that herbaceous species tend to adopt better adaptive strategies such as constructing low‐cost above‐ground tissue, shortening life spans and forming underground nutrient storage organs, all of which allow them to be free from environmental stresses such as freeze or aridity (Hu et al. 2010; Zanne et al. 2014). Besides, compared with their woody counterparts, herbaceous vines usually possess more effective root pressures in order to minimise the effects of drought‐ or freeze‐induced embolisms (Ewers et al. 1997; Ewers and Fisher 1991). In contrast, woody climbers are more liable to be dominated by climate. Often equipped with large xylem vessels, they are made more susceptible to droughts or coldness, and therefore more sensitive to harsh conditions and climatic variations (Jiménez‐Castillo and Lusk 2013). As a result, only a minority of woody members have successfully survived in extratropics relying on much more complicated mechanisms to cope with freezing, droughts and larger amplitudes of climatic fluctuations (Jiménez‐Castillo and Lusk 2013), and most of them are restricted to relatively small geographical ranges (see Figure S2, range comparations for the three subtypes of growth forms). Collectively, high proportions of broad‐niched generalists within herbaceous groups lead to higher similarity among local species assemblages, which ultimately invokes relatively even family age patterns of vines.

## 5 Conclusion

One of the most important findings in our study is that the relationship between MFA of climbing plants and temperature along the latitudinal gradient in China is contrary to the prediction of TNC but consistent with that of OTT. Given the negative relationships found in our study as well as other available empirical studies, it can be inferred that the TNC hypothesis may not necessarily provide explanations for all LDG‐linked patterns, at least not for the climbing taxa in China. Indeed, we do not insist that the underlying mechanisms proposed by TNC are noneffective, but we do highlight here that there may exist some places on Earth where other historical OTT‐linked processes might have played a more important role than TNC along latitudinal gradients. To verify the generality of these results, further comparative studies are critically needed to be conducted on regional scales elsewhere concerning the MFA latitudinal gradients for non‐tree taxa.

## Author Contributions

**Mingfei Zhao:** conceptualization (equal), data curation (equal), investigation (equal), methodology (equal), visualization (equal), writing – original draft (equal). **Hongbo Li:** writing – original draft (equal), writing – review and editing (equal). **Yuhang Wang:** writing – original draft (equal), writing – review and editing (equal). **Yuan Jiang:** writing – review and editing (equal). **Muyi Kang:** writing – review and editing (equal). **Kaixiong Xing:** funding acquisition (lead), project administration (equal), supervision (equal), visualization (lead), writing – review and editing (equal).

## Conflicts of Interest

The authors declare no conflicts of interest.

## Supporting information


**Data S1.** Supporting Information.

## Data Availability

The data that support the findings of this study are available in the Supporting Information—S1 of this article.
