# Supplementary material for: ‘Out of the Tropics’ Sheds Light on Latitudinal Gradients in Clade Ages of Climbers, China
Source: Ecol Evol. 2025 Apr 18;15(4):e71324. doi: 10.1002/ece3.71324 (PMC12008040; doi:10.1002/ece3.71324)
Supplement: Supplementary file 1 — Data S1. Supporting Information. [file ECE3-15-e71324-s001.docx]

## Electronic supplementary materials

**Figure S1** The comparison of the residual autocorrelations in multiple ordinary least squares (OLS) regression and spatial simultaneous autoregressive error (SAR_err_) models based on Moran’s *I* index for all climbers and three subgroups of growth form, which showed that the SAR_err_ models could effectively eliminate the spatial autocorrelation.


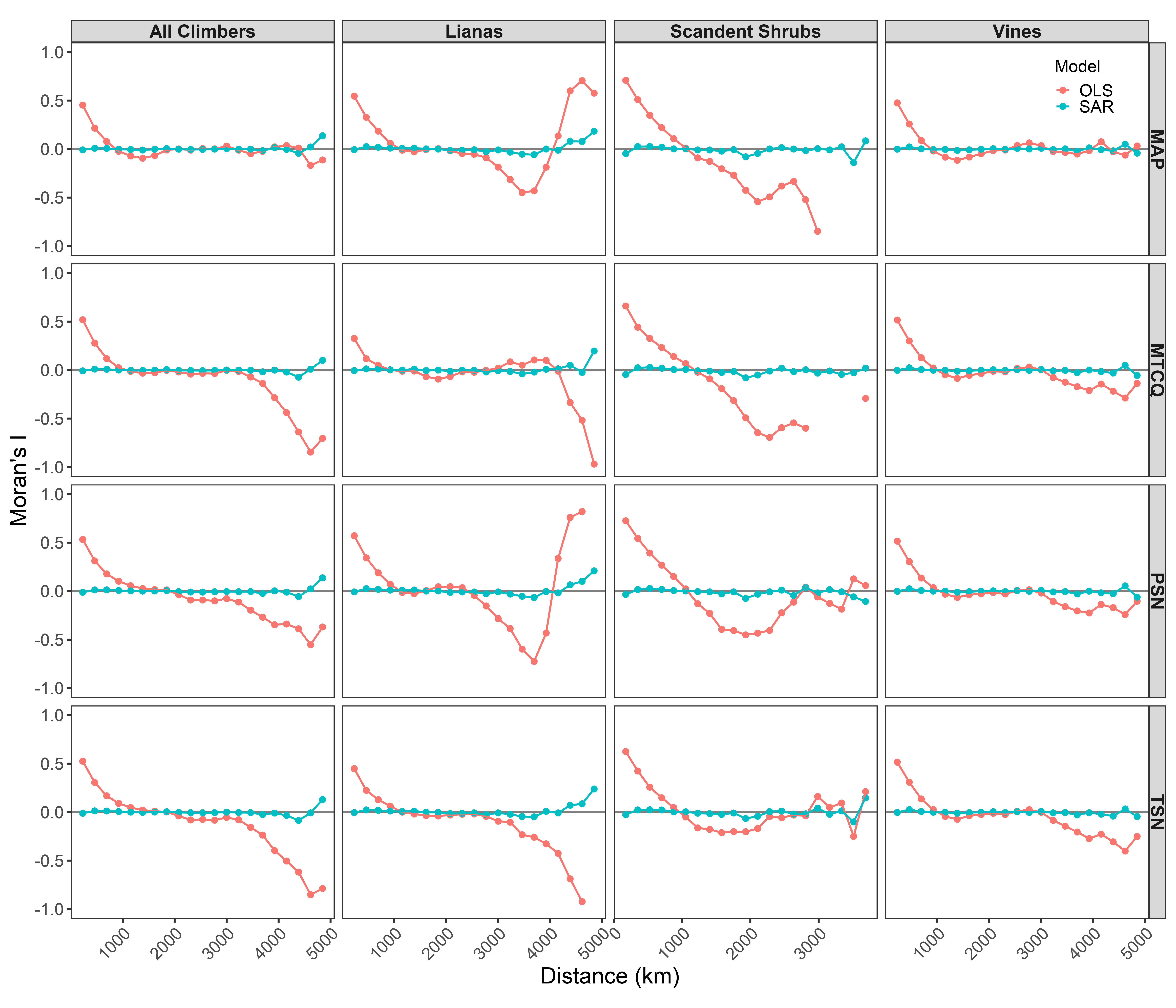


**Figure S2** Range data( the number of 100km×100km grids per species covers) for three climbing growth forms are presented as violin plots overlaid with box plots using pairwise Wilcoxon rank sum test. In the box plots, the points (white) and hinges represent the 25th, 50th and 75^th^ percentiles, the white lines mark the mean values, and the whiskers represent 95% confidence intervals. In the violin plots, the thickness of the violin polygon corresponds to the empirical distribution of the observations. Number (of species) in parentheses shows the sample size.


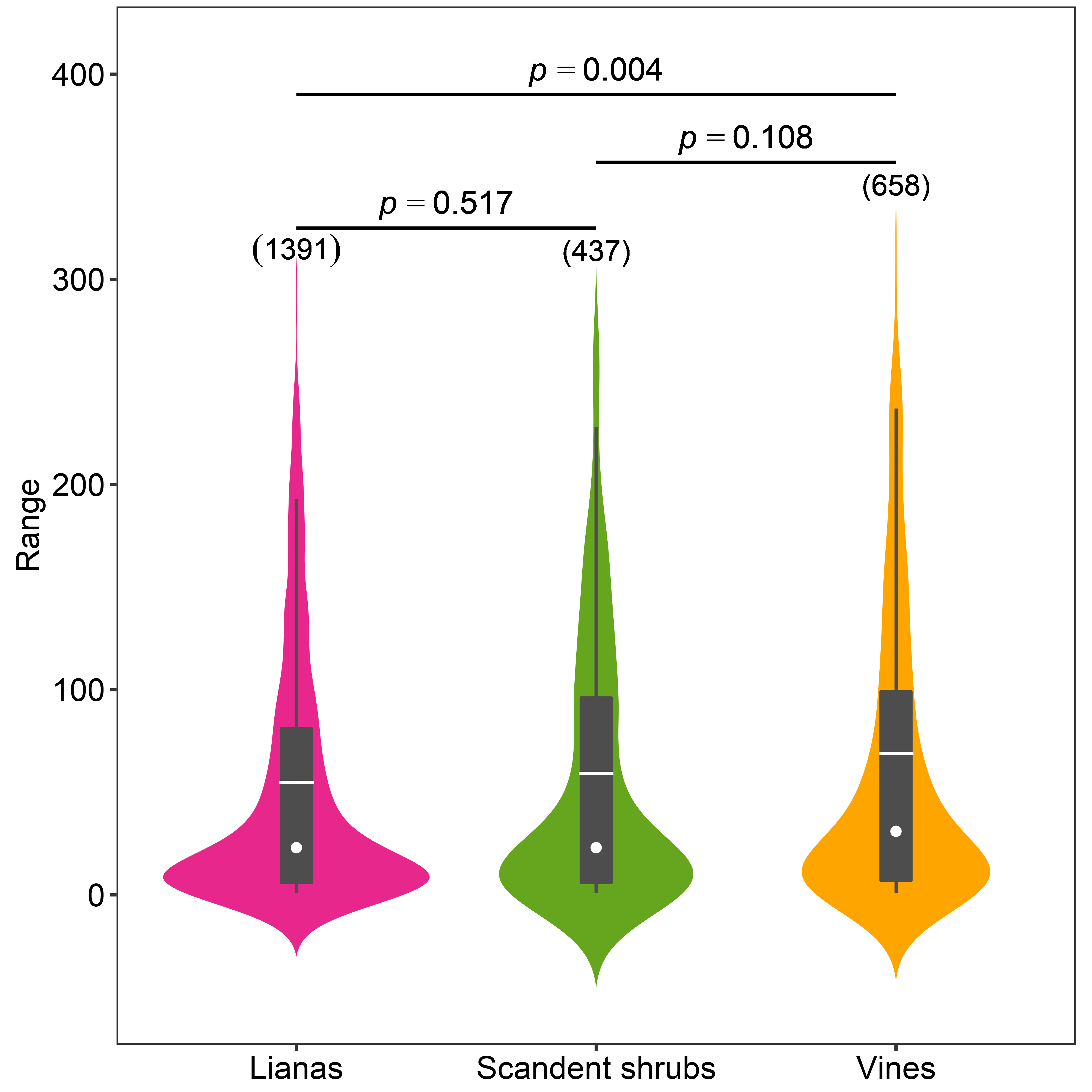


**Table S1** List of age of families which were obtained from Zanne *et al*. (Zanne *et al.*, 2013). Climber types were referred by the description for each species from Flora of China (http://www.iplant.cn/foc).

| **Family** | **Growth form** | **Age(myr)** |
| --- | --- | --- |
| Acanthaceae | Lianas, Vines | 36.7 |
| Actinidiaceae | Lianas | 68.4 |
| Amaranthaceae | Scandent Shrubs | 38.4 |
| Anacardiaceae | Lianas | 59.1 |
| Ancistrocladaceae | Lianas | 49.2 |
| Annonaceae | Lianas, Scandent Shrubs | 45.5 |
| Apocynaceae | Lianas, Scandent Shrubs, Vines | 37.6 |
| Araceae | Lianas | 123.2 |
| Araliaceae | Lianas, Scandent Shrubs | 65.5 |
| Arecaceae | Lianas | 115 |
| Aristolochiaceae | Lianas, Vines | 121 |
| Asparagaceae | Vines | 70.7 |
| Asteraceae | Lianas, Scandent Shrubs, Vines | 45.7 |
| Bignoniaceae | Lianas | 24.8 |
| Boraginaceae | Scandent Shrubs | 55.8 |
| Campanulaceae | Vines | 53 |
| Cannabaceae | Vines | 47.7 |
| Capparaceae | Lianas, Scandent Shrubs | 71.2 |
| Caprifoliaceae | Lianas | 65.6 |
| Celastraceae | Lianas, Scandent Shrubs | 67.7 |
| Colchicaceae | Vines | 84.2 |
| Combretaceae | Lianas | 65.5 |
| Commelinaceae | Vines | 73.9 |
| Connaraceae | Lianas | 26.8 |
| Convolvulaceae | Lianas, Vines | 54.1 |
| Cornaceae | Scandent Shrubs | 65.4 |
| Cucurbitaceae | Lianas, Vines | 68.4 |
| Dichapetalaceae | Lianas | 18.9 |
| Dilleniaceae | Lianas | 51.9 |
| Dioscoreaceae | Vines | 105.2 |
| Elaeagnaceae | Scandent Shrubs | 56.4 |
| Ericaceae | Scandent Shrubs | 62.1 |
| Erythropalaceae | Lianas | 41.9 |
| Euphorbiaceae | Lianas, Scandent Shrubs | 99.3 |
| Fabaceae | Lianas, Scandent Shrubs, Vines | 69.1 |
| Flagellariaceae | Lianas | 61.7 |
| Gelsemiaceae | Lianas | 33.9 |
| Gentianaceae | Vines | 29.6 |
| Gesneriaceae | Lianas | 37.4 |
| Grossulariaceae | Scandent Shrubs | 76.2 |
| Hernandiaceae | Lianas | 71.2 |
| Hydrangeaceae | Lianas, Scandent Shrubs | 63.8 |
| Icacinaceae | Lianas | 58.7 |
| Lamiaceae | Scandent Shrubs | 32.9 |
| Lardizabalaceae | Lianas | 97.1 |
| Loganiaceae | Lianas | 34.4 |
| Malpighiaceae | Lianas, Scandent Shrubs | 69.8 |
| Malvaceae | Lianas, Scandent Shrubs | 69.1 |
| Melastomataceae | Scandent Shrubs | 59.8 |
| Menispermaceae | Lianas, Vines | 67.1 |
| Moraceae | Lianas, Scandent Shrubs | 54.8 |
| Nepenthaceae | Vines | 75.7 |
| Nyctaginaceae | Lianas | 34.7 |
| Oleaceae | Lianas, Scandent Shrubs | 39.4 |
| Opiliaceae | Lianas | 32.7 |
| Orchidaceae | Vines | 111.4 |
| Orobanchaceae | Scandent Shrubs | 35.5 |
| Pandanaceae | Lianas | 34.1 |
| Papaveraceae | Vines | 113 |
| Passifloraceae | Lianas, Vines | 59.8 |
| Phyllanthaceae | Lianas | 98.3 |
| Piperaceae | Lianas, Vines | 31.2 |
| Plantaginaceae | Vines | 31.7 |
| Polygalaceae | Lianas, Scandent Shrubs | 60.1 |
| Polygonaceae | Vines | 54 |
| Primulaceae | Scandent Shrubs | 54 |
| Ranunculaceae | Lianas, Vines | 105.4 |
| Rhamnaceae | Scandent Shrubs | 76.2 |
| Rosaceae | Scandent Shrubs, Vines | 82.3 |
| Rubiaceae | Lianas, Scandent Shrubs, Vines | 56.9 |
| Rutaceae | Lianas, Scandent Shrubs | 45.5 |
| Sabiaceae | Lianas | 91.7 |
| Santalaceae | Lianas | 17 |
| Sapindaceae | Vines | 66.3 |
| Schisandraceae | Lianas | 105.4 |
| Smilacaceae | Lianas, Scandent Shrubs, Vines | 80.6 |
| Solanaceae | Scandent Shrubs, Vines | 47 |
| Stemonaceae | Vines | 56.3 |
| Urticaceae | Lianas, Vines | 56.2 |
| Vitaceae | Lianas | 111.9 |
